# Self‐testing, communication and information technology to promote HIV diagnosis among young gay and other men who have sex with men (MSM) in Brazil

**DOI:** 10.1002/jia2.25116

**Published:** 2018-07-22

**Authors:** Raquel B De Boni, Nena Lentini, Ana CFS Santelli, Aristides Barbosa, Marly Cruz, Trista Bingham, Vanda Cota, Renato Girade Correa, Valdiléa G Veloso, Beatriz Grinsztejn

**Affiliations:** ^1^ Evandro Chagas National Institute of Infectology (INI) Oswaldo Cruz Foundation (Fiocruz) Rio de Janeiro Brazil; ^2^ Division of Global HIV and TB (DGHT) Centers for Disease Control and Prevention (CDC) Country Office in Brasília Brasília Brazil; ^3^ Sérgio Arouca National School of Public Health (ENSP) Oswaldo Cruz Foundation (Fiocruz) Rio de Janeiro Brazil; ^4^ Division of Global HIV and TB (DGHT) Centers for Disease Control and Prevention (CDC) Atlanta GA USA; ^5^ IST HIV/AIDS and Viral Hepatitis Department (DIAHV) Ministry of Health of Brazil Brasília Brazil

**Keywords:** men who have sex with men, key and vulnerable populations, self‐testing, HIV, mobile applications, Brazil

Worldwide, key populations (KP), including gay and other men who have sex with men (MSM), are subject to human rights violations, criminalization, stigma and discrimination 1, 2. These socio‐structural factors are crucial to understand the low HIV testing uptake in many countries, as MSM may fear or may have experienced lack of privacy, confidentiality breaches and healthcare staff mistreatment 3. In Brazil, MSM report a low frequency of HIV testing despite higher estimated HIV prevalence (9.4% among 18 to 24 year olds; 19.8% among those 25 years and older 4), compared with 0.6% among the general population 5. HIV self‐testing (HIVST) is currently recommended by the World Health Organization to help reduce gaps in HIV diagnosis, especially for KP 6. Furthermore, HIVST has been highly accepted and accurate 7, 8, with oral tests being preferred over blood tests 9.

With the need to expand HIV diagnosis options for MSM, especially among young MSM, a committed team of governmental, research and non‐governmental organizations in Curitiba, Brazil launched and evaluated a multi‐component implementation science project from February 2015 to February 2017 to improve HIV outcomes for MSM. This project, called *A Hora É Agora* (The Time is Now) 10, implemented a multi‐pronged approach to increase HIV testing and linkage to care among MSM. The most innovative of the project's components was a web‐based platform and associated mobile application designed to provide HIV prevention information, allow for self‐assessment of risk, and deliver HIVST packages to eligible individuals (males, 18 years old and up, resident in Curitiba, with negative/unknown HIV status) upon request 11. Each HIVST package contained two oral‐fluid test kits, instructions for use and interpretation of HIVST results, a supply of condoms and lubricant, and information on confirmatory testing. Options for receiving the HIVST kits included either home delivery by mail or pick‐up at a government‐sponsored pharmacy.

A centerpiece of the project was a communications plan tailoring dynamic visuals with printed and virtual messaging to appeal to the target population; an attractive, online instructional video for HIVST users [https://www.ahoraeagora.org]; and frequent in‐person outreach events in places where MSM socialize in Curitiba. The project maximized the use of social media to reach out to and to engage young men in HIVST. Facebook and gay online sites such as ManHunt and Grindr played a key role in disseminating HIV testing messages. Mobile tools, such as WhatsApp and other freeware instant messaging applications boosted communications between users and project staff, including health system navigators for linkage to care. Working to ensure outreach to these groups, organizations involved in project implementation partnered with gay and MSM‐friendly establishments such as saunas, movie theatres, cafes, and bars to further disseminate HIVST information.

With an initial goal to distribute 1000 test kits per year, the project quickly exceeded all expectations with 7352 HIV self‐test requests over 24 months (Figure 1).

**Figure 1:**
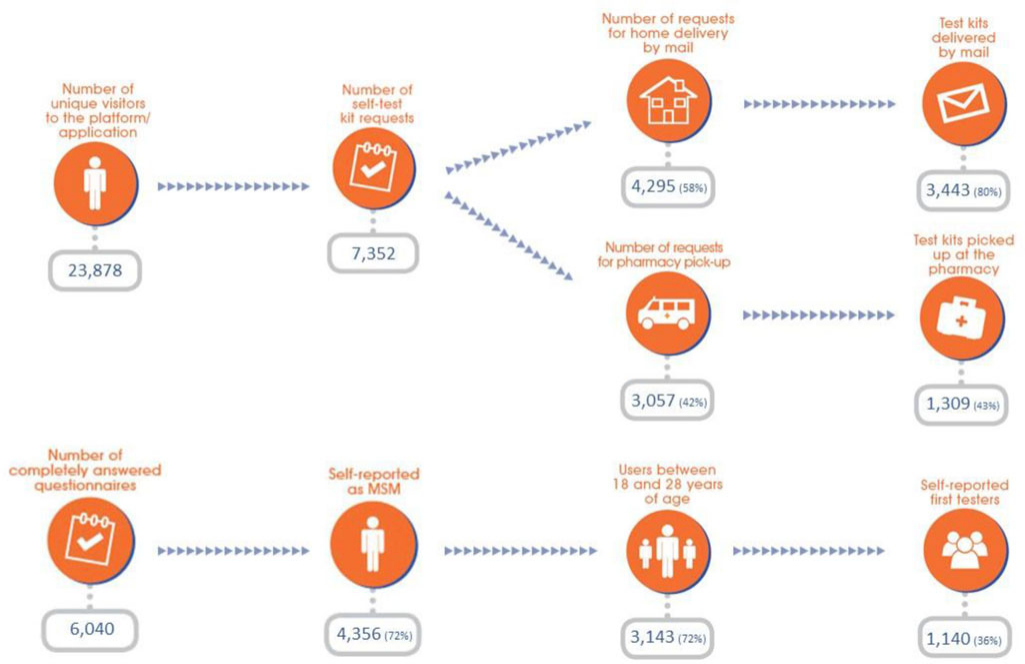
Web‐based and mobile platform, HIV self‐testing uptake and men who have sex with men (MSM) testing for the first time. A Hora É Agora Project, Curitiba (Brazil), 2015 to 2017.

Beyond the high demand, the project was able to reach a large percentage (31%) of MSM who had never tested before, with those between 18 and 28 years old reporting a higher percentage of first‐time testers (36%), than those 29 years or older (18%). Of the 4356 MSM who completed the online risk survey, 72% were 18 to 28 years old, showing how innovative strategies can address the common challenge of increasing youth access to healthcare 12.

From the design phase through programme implementation, MSM's anonymity, privacy and targeted messaging formed the critical pillars of this initiative – confirmed by users’ preferred choice of delivery by mail (58%). Although this option required a valid address, users were able to use any name and any address where they were most comfortable receiving the test kit. To ensure privacy, the HIVST kits were mailed in a plain cardboard box with no indication of its contents.

The availability of confirmatory testing and health navigation options for those who self‐reported a positive screening test were critical components of the comprehensive project. Although not mandatory, 34 individuals voluntarily reported a reactive HIVST result on the project website. Understanding HIVST as a screening strategy, 44 sought confirmatory testing in the project‐recommended health unit. Of these, 40 accepted linkage to HIV services support by peers and health system navigators, another component of the project that assisted new patients entering Brazil's decentralized health system and the cascade of care.

## 1 Improving upon Curitiba's model

With essential adjustments to Curitiba's promising web‐based HIVST model, we have recently expanded the project to São Paulo, Brazil, the largest metropolitan area in South America (population 12 million) with the highest concentration of people living with HIV and the majority of new infections in Brazil.

The relatively high cost of mail delivery and the lower observed uptake of pharmacy‐based HIVST pick‐ups sparked creative thinking among organizations responsible for expansion to São Paulo. As a result, automated HIVST dispensers will be installed in target areas in both Curitiba and São Paulo, with a focus on venues that are open 24/7 and near gathering points of gay and other MSM. Users requesting HIVST kits via web‐based and mobile platforms will receive a randomly generated, four‐digit code to be entered into strategically placed dispensing machines that distribute tests from individual cabinets. These self‐service dispensers are expected to be a key option for reduced costs and increased ease of access. The project expects to dispense 10,000 tests in São Paulo by September, 2018.

Communication and information technologies have enhanced HIVST delivery in Brazil and show promise in attracting young gay and other MSM who value anonymity and privacy in accessing HIV services for diagnosis and subsequent treatment for positive cases. The success of Brazil's web‐based HIVST platform may translate well to other countries that struggle to serve gay and other MSM in the context of societal and self‐stigma, narrowing inequalities in test access. As we embark on the expansion of this programme to São Paulo and beyond, we anticipate learning additional lessons on how to encourage systematic reporting of results, expand access to other key populations, reduce costs, and improve sustainability while achieving epidemic control.

## Competing interests

All authors declare that they have no significant competing financial, professional, or personal interests that might have influenced the performance or presentation of the work described in this manuscript.

## Authors’ contributions

RBB, ABJ, VGV, MC, and BG participated in study design. RBB, ABJ, NL, MC, RGC and VC were involved in planning and supervision. RBB, VGV, RGC and BG analysed the data. RBB, NL, ACFSS and TB wrote this paper with input from all authors. All authors approved the final version of the manuscript and are responsible for all aspects of this study, thus ensuring its accuracy and integrity.

## Funding

This publication was supported by the Cooperative Agreement Number NU2G GH001152, funded by the United States President's Emergency Plan for AIDS Relief (PEPFAR) through the Centers for Disease Control and Prevention (CDC). Its contents are solely the responsibility of the authors and do not necessarily represent the official position of the funding agencies.
